# Literacy: A lever for citizenship?

**DOI:** 10.1007/s11159-023-09998-6

**Published:** 2023-05-10

**Authors:** Anna Robinson-Pant

**Affiliations:** UNESCO Chair in Adult Literacy and Learning for Social Transformation, School of Education and Lifelong Learning, University of East Anglia, Norwich, UK

**Keywords:** Adult literacy, Citizenship education, Citizenship, Ethnography, UNESCO

## Abstract

Within citizenship education, literacy is often promoted in a narrow functional sense of skills for civic engagement or is used synonymously with “knowledge” to refer to an awareness-raising process around rights. Through an analysis of evolving models of citizenship, this article moves beyond literacy *for* citizenship to consider the ways in which literacy learning can emerge *through* active citizenship. Drawing on published ethnographic studies of literacy in everyday life to analyse both the symbolic and instrumental meanings of literacy in specific contexts, the author introduces a social practice lens on literacy and citizenship. She explores the pedagogical implications for literacy within citizenship education, particularly in relation to informal learning of “real literacies”, critical digital literacy to distinguish “fake news” and literature as a way of entering someone else’s experiences. UNESCO’s current vision for global citizenship education as nurturing empathy and understanding between peoples implies that literacy providers need to recognise participants as not only consumers, but as co-constructors of texts.

## Résumé

L’alphabétisation, un levier de la citoyenneté ? – Dans le domaine de l’éducation à la citoyenneté, soit l’alphabétisation fait souvent l’objet d’une promotion dans le sens fonctionnel étroit de l’acquisition de compétences favorisant l’engagement civique, soit elle est employée comme synonyme de « savoir » renvoyant à un processus de sensibilisation lié aux droits. En analysant les modèles de citoyenneté qui évoluent, cet article va au-delà de l’alphabétisation *pour* la citoyenneté et examine les façons dont l’alphabétisation peut apparaître *par le biais* de la citoyenneté active. L’autrice s’appuie sur des études ethnographiques sur l’alphabétisation dans la vie quotidienne pour analyser les significations symboliques et instrumentales de l’alphabétisation dans des contextes spécifiques et éclaire l’alphabétisation et la citoyenneté du point de vue des pratiques sociales. Elle examine les implications pédagogiques pour l’alphabétisation dans le cadre de l’éducation à la citoyenneté, notamment en ce qui concerne l’apprentissage informel d’« alphabétisations réelles », l’alphabétisation numérique critique pour distinguer les « infox » et la littérature comme moyen de pénétrer dans l’expérience d’un autre. La vision actuelle de l’UNESCO selon laquelle l’éducation à la citoyenneté mondiale nourrit l’empathie et favorise la compréhension entre les peuples implique que les prestataires d’alphabétisation ne considèrent pas les participants uniquement comme des consommateurs, mais qu’ils voient en eux des co-constructeurs de textes.

## Introduction

The relationship between literacy and citizenship is usually seen as a “given” and as unproblematic – including the assumption that literacy is an essential prerequisite for active citizenship. But do we really understand why, when and how literacy is connected with citizenship? All too often, the term “literacy” is used in a metaphorical sense to mean “knowledge” or “learning” (as in the phrase “emotional literacy”), thus obscuring the ways in which reading, writing, constructing and decoding diverse texts may support – or possibly undermine – citizenship. This article sets out to explore the relationship through a sharper lens, starting from the understanding that there is neither only one kind of literacy nor only one model of citizenship.

The *5th Global Report on Adult Learning and Education* (*GRALE 5*) (UIL 2022a) was launched at the Seventh International Conference on Adult Education (CONFINTEA VII), held 15–17 June 2022 in Marrakech. The report’s thematic focus on “Citizenship education: empowering adults for change” signals an important opportunity to reflect on how exactly literacy can contribute to strengthening the spirit of citizenship. Compared to the *GRALE 4* survey (UIL 2019) three years earlier, the *GRALE 5* survey data reveal “an increasing policy attention to citizenship education” around the world, demonstrating that citizenship education is no longer a marginal element of adult learning and education (ALE) curricula: “Close to three quarters (74%) of countries indicated that they are developing or implementing policies in relation to citizenship education” (UIL 2022a, p. 19). However, the report goes on to note that the concept of citizenship education is understood in quite different terms from country to country, sometimes being equated more narrowly as raising awareness about environmental issues, rather than, for instance, encompassing human rights and media literacy. These differences indicate how the model of citizenship taken up by political leaders will influence approaches to education for citizenship – and emphasise the importance of looking at changing understandings of citizenship, over time and around the world.

In terms of the interrelationship between literacy and citizenship education, Paulo Freire’s work highlighted the ways in which literacy has been seen as both an obstacle and an opportunity to engage in active citizenship at differing times throughout history, and his work is a key reference both in the *GRALE 5* report (UIL 2022a) and in wider policy discussions. He links literacy directly with politics: “I always saw teaching adults to read and write as a political act, an act of knowledge, and therefore a creative act” (Freire and Macedo 1987, p. 34). His argument for critical and liberating dialogue is developed in relation to the opposite – “domesticating” narrative education, where the teacher’s task is “to ‘fill’ the students with the contents of his narration – contents which are detached from reality” (Freire 1996 [1970], p. 53). However, Freire sees this conventional “banking” educational approach as equally “political”, in that “to substitute monologue, slogans and communiques for dialogue is to attempt to liberate the oppressed with the instruments of domestication” (ibid., p. 47). What comes through so strongly in Freire’s work is his attention to *which* words are being decoded: “the word is more than just an instrument which makes dialogue possible” (ibid., p. 68). His discussion of “true” words centres on the relationship of literacy to citizenship and action: “when a word is deprived of its dimension of action, reflection automatically suffers as well; and the word is changed into idle chatter …” (ibid.).

Freire has been critiqued by Brian Street for over-stating the importance of literacy within adult education, and thus promoting the illiterate/literate divide.^1^ For instance, Freire continually emphasised the significance of literacy as learning to “communicate graphically”, thus facilitating “an attitude of creation and re-creation, a self-transformation producing a stance of intervention in one’s context” (Freire and Macedo 1998, p. 86). Freire’s writing does however provide an insight into the ways in which texts and reading practices can sometimes construct social hierarchies and exacerbate inequalities in voice and identities, thus constraining opportunities for active citizenship.

In order to explore how literacy can become a lever for citizenship, I will begin with a historical account of the development of different citizenship models, identifying influences on the changes that have taken place. This is an essential starting point for investigating various approaches to citizenship education, particularly the intended outcomes and purposes. I will then explore where literacy comes into this picture, drawing on a “situated” (Barton et al. 2000) or “social practice” (Street 1995) conceptualisation of literacy in order to analyse the implications for citizenship education. Through pulling together literature on citizenship, education and literacy, this article aims to provide a conceptual map which can be used to explore the objectives and scope of literacy programmes in relation to citizenship outcomes. I will conclude with some ideas that could help educational providers to strengthen the connections between literacy and citizenship in future.

## Citizenship: a changing concept

[C]itizenship has throughout history been a legal and political status accorded by the state to the individual and a bond of loyalty owed by the individual to the state (Heater 2004, p. 194).

The concept of citizenship can be traced back to ancient Greece, where it evolved around commitment to the wellbeing of the *polis*. Derek Heater (2004) explains that by the 8th century, the kingdom or the tribe was no longer the main unit for governance in Greece, but the polis – defined as “a compact community dominated by a relatively small and ethnically cohesive group” (ibid., p. 1). The bounded nature of the *polis* and the shared identity/language of the population (though foreigners/outsiders did work for the *polis*) meant that the two-way relationship between the individual and the state was relatively straightforward. As Ralph Dahrendorf (1996) suggests, “citizenship describes the rights and obligations associated with membership in a social unit, and notably with nationality … who can be a member and who cannot” (ibid., p. 31). Within the ancient Greek model of citizenship, the purpose of education was to strengthen the bond between the individual and the state, as indicated in Aristotle’s *Politics*, 4th century BC: “the polis … is an aggregate of many members; and education therefore is the means of making it a community and giving it unity” (Aristotle 1948, 1263b, cited in Heater 2004, p. 2).

Over the centuries, this unitary model of citizenship – based on an assumption of shared identities, borders and values – has been strongly challenged. Melissa Williams identifies how the original concept of citizenship has been complicated by some of the tensions emerging in societies:

most of our current understandings of citizenship are based on the historic convergence of boundaries of citizenship (territorial, cultural/national/linguistic, institutional and moral) that are now pulling apart (Williams 2003, p. 209).

Writing on “multiple citizenship education”, Heater (2004, p. 194) suggests that significant challenges have emerged from “above”, in terms of the creation of transnational institutions that offer citizenship status like the EU “or belief in the concept of world [or global] citizenship, with a moral code that transcends the obligations of state law” (ibid.). Such developments and processes of globalisation have brought into question the idea that the nation-state is the main unit of collective identity. The role of the nation-state, and its authority to determine citizenship, has also been challenged “from below” by ethnic and cultural minority groups with distinct identities and languages who challenge “the conflation of [the political] state and [the cultural] nation” (ibid., p. 195).

The abstract concept of the citizen dominant in many political and educational discourses has been strongly contested by feminist writers who argue that it “masks its deeply undemocratic social relations and institutions” (Arnot and Dillabough 2000, p. 4). Discussing the ways in which the “concept of citizen has been constructed as male and the ‘other’ as female” (ibid., p. 22), they suggest that this binary has been intensified by the separation between public and private spheres. The identification of the public sphere with men, and the private with women, meant that “citizenship”, being located in the public sphere, often excluded/excludes women. Through digital communication, this dichotomy between public and private spheres is now being challenged to an extent in virtual spaces. New kinds of community are being constructed through social media and conventional media, involving values and practices similar to citizenship – such as voting by the public on TV shows (Cremin 2012).

These diverse influences raise important questions about citizenship, inclusion and exclusion in our world today: what are the boundaries of society? “Which groups belong and which do not?” (Bulmer and Rees 1996, p. xv). Is it possible to be a world citizen, as the world is not a state? (Heater 2004, p. 195, on the “semantic confusion” of the term “world citizenship”). New conceptualisations of citizenship are moving away from that of a singular identity and a simple two-way relationship between state and individual. Williams proposes that “instead of a model of citizenship-as-identity, we should move toward an idea of citizenship as membership in a community of shared fate” (Williams 2003, p. 209). She argues that for “shared identity”, people need a reason to identify with a particular political community, based on their culture or values, whereas “shared fate” is simply a recognition that our futures are entangled with one another. The concept of global citizenship, which underpins the current approach of the United Nations Educational, Scientific and Cultural Organization (UNESCO) to citizenship education, seems to draw on a similar idea of “shared fate” – particularly in relation to the future of our planet.

Much of the recent writing on citizenship education appears to be centred on a necessary connection between democracy, equality and citizenship, which may date back to Thomas Humphrey Marshall’s (1950) seminal historical account of the development of citizenship in England (which *GRALE 5* also draws upon). His analysis of the tensions between social class and citizenship is based on the idea that citizenship is “a principle of equality” (Marshall 1950, p. 33), yet its growth coincided in England “with the rise of capitalism, which is a system, not of equality, but of inequality” (ibid., p. 29). In today’s context of increasing global mobility, conflicts and displaced peoples, I suggest that citizenship is not always used as “a principle of equality” – and, like literacy, could be regarded as a “double-edged sword”. Although I have approached this section as a historical account, it should be noted that the early assumptions of a unitary shared identity and bounded community still inform notions of citizenship in many country contexts today. It is important to engage with and recognise the different understandings of citizenship outlined in this section, as they influence approaches to education and literacy taken by nation-states and international organisations – such as whether the aim is for people to assimilate into a specific society, rather than a recognition of multiple cultural and intercultural identities.

## Citizenship education: towards multiple, global and transformative approaches

Returning to ancient Greece, we find that education was considered integral to citizenship, in order “to induct the individual into that status and to clinch that bond”, as Heater (2004, p. 194) suggests. Aristotle defined the three objectives of civic education as follows:

It is the responsibility of citizens to contribute to the cohesiveness and therefore the stability of the state … The second objective was the specific and practical learning of the citizen’s duties … The third objective was to teach young citizens their rights, social, legal and political (Heater 2004, p. 2, referring to Aristotle 1948).

These three aspects are reflected in subsequent models of citizenship education, particularly the emphasis on rights and duties to contribute to the stability of the state. The “practical learning” could be related to the concept of “active citizenship” which underpins many current policy initiatives, including those analysed in *GRALE 5* (UIL 2022a). The attention to preparing “young citizens” in Aristotle’s third objective is significant, since citizenship education in schools was originally framed in terms of the rights for an adult to be prepared for citizenship – rather than the rights of children as citizens (Marshall 1950).

These continuities can be identified in citizenship education approaches in many parts of the world today – particularly within civic education. However, the evolving conceptualisation of citizenship discussed in the previous section has raised new dilemmas around what citizenship education is for and about. The broader and more complex notion of multiple citizenship has led us to ask: In a diverse plural society, whose values should we be teaching? “What sort of identity should a program of civic education inculcate in citizens?” (Williams 2003, p. 215) There have been concerns around how or whether citizenship can be taught – the suggestion that it is instead “caught not taught” (Davies 2012, p. 37), for instance, through informal learning and active engagement in civic responsibilities. Above all, there are fears that teaching a cosmopolitan ethic or global citizenship could reduce pupils’ loyalty to the nation-state.

Martha Nussbaum addressed these issues directly when she made the case for world citizenship education, developing pedagogical principles and curriculum objectives from these cosmopolitan ideas around citizenship (Nussbaum 1997). She proposed that world citizenship education involves the development of three capacities. Drawing on Socrates’ notion of the “examined life”, she argues that the first capacity involves: “critical examination of oneself and one’s traditions” (Nussbaum 2002, p. 293) and development of critical, analytical skills. The second capacity relates to the ability of citizens

to see themselves as not simply citizens of some local region or group but also, and above all, as human beings bound to all other human beings by ties of recognition and concern (ibid., p. 295).

Significantly, Nussbaum relates this capacity to teaching “involving non-Western cultures” and “internal minorities” (ibid., p. 296), referring in her article on the importance of liberal arts education to Americans as being “tremendously ignorant of other nations of the world” (ibid.). This aspect of citizenship education has particular resonance with the growing policy attention to Indigenous learning, worldviews and knowledges.

The third capacity essential to world citizenship is what Nussbaum terms “the narrative imagination” (ibid., p. 299). She explains this as

the ability to think what it might be like to be in the shoes of a person different from oneself, to be an intelligent reader of that person’s story, and to understand the emotions and wishes and desires that someone so placed might have (ibid.).

Such a stance, she suggests, is not uncritical – similar to when we read about a character in a novel and make judgements about their behaviour and beliefs. This is where education in literature and the arts is important, Nussbaum argues – to begin to understand meanings of speech and writing in the context of “that person’s history and social world” (ibid., p. 299). She highlights the dangers of the “moral imagination” becoming “lazy” through refusing sympathy to “people who look different”. This emphasis on empathy and exercising “the muscles of the imagination” (ibid., p. 300) through reading and discussion points to an important element of literacy education within universities, a pedagogical approach which could be applied to basic adult literacy programmes too.

By emphasising the importance of both global and local ties, Nussbaum argued strongly that it was not a question of either/or with regard to national and/or world citizenship – but that through cosmopolitan education, we can learn more about ourselves and our specific nation. This stance reflects current directions in global citizenship education: the starting point that global citizenship is not an alternative to, but rather adds value to national citizenship. As Ulrike Hanemann outlines, citizenship is “active”, “critical” and “diverse”, not limited to action in one sphere only:

The overall goal of *global citizenship education* is to empower learners to engage and assume active roles both locally and globally to face and resolve challenges (Hanemann 2019, p. 4; emphasis in original).

The three capacities identified by Nussbaum are echoed in *GRALE 5:*

global citizenship education should be transformative, building the knowledge, skills, values and attitudes that learners need to be able to contribute to a more inclusive, just and peaceful world (UIL 2022a, p. 122).

It is notable that literacy is not mentioned explicitly here in relation to the skills required – yet there is still an assumption that citizenship is closely connected with literacy, as the main vehicle for conveying citizenship knowledge and practising citizenship. Ernest Gellner’s belief that “literacy is the minimum requirement for the exercise of citizenship” (Heater 2004, p. 198, referring to Gellner 1983) is shared by many, and the next section explores why.

## Taking a social practice lens on literacy and citizenship

*GRALE 5* concludes that literacy is “arguably the primary precondition for democratic citizenship and demonstrably a social practice with great transformative potential” (UIL 2022a, p. 161). So how exactly does literacy come into citizenship education? Freire’s literacy work in the early 1960s in Brazil has since inspired many adult educators – particularly his idea of literacy as facilitating a process of conscientisation leading to reflection and social action. Nelly Stromquist’s (1997) research explored the ways in which the Freirean-influenced Brazilian literacy programme *Movimeno de Alfabetizacao de Jovens e Adultos* (MOVA) set out to link literacy and citizenship. This programme aimed

to enable the large number of disenfranchised and passive poor to see themselves as individuals with rights (and duties) upon the state, to position themselves as citizens with legitimate demands for social change and for a life that recognised their claims as individuals regardless of social class, race, and gender differences (Stromquist 1997, p. 1).

The focus on people becoming aware of their rights and duties takes us back to Aristotle’s objectives of civic education discussed earlier, and the notion of “multiple identities” (class, race and gender) relates to the move away from citizenship as based on a unitary identity. Above all, the MOVA programme aimed to facilitate an educational process of critical reflection on society and the individual’s role within that society. But how did this process connect to literacy, as opposed to other kinds of learning within radical adult education?

In its *GRALE 5 Executive Summary*, the UNESCO Institute for Lifelong Learning (UIL) reports that “[t]here is ample evidence that literacy learning correlates with positive citizenship outcomes” (UIL 2022b, p. 9), citing benefits such as improved self-esteem, empowerment, creativity and critical reflection. Similar outcomes were reported in the MOVA study too, though Stromquist notes that simply getting out of the house and mixing with other women in a public space could have contributed to confidence building, rather than “literacy skills per se” (Stromquist 1997, p. 139). She suggests that “[t]o insist on a pure attribution to literacy skills sets up false conditions” (ibid.). This warning against adopting what Street (1984) refers to as an “autonomous” model of literacy draws attention to the common assumption that literacy learning can convey certain cognitive and social benefits regardless of context. Given the diverse approaches to citizenship and citizenship education around the world, we need to consider what literacy means in different contexts as a basis for exploring assumptions about literacy in citizenship discourses.

Turning to another seminal literacy research study, I will now take an “ideological” perspective on literacy (Street 1984) through ethnographic research conducted by the Moroccan Literacy Project – a collaboration between Mohamed V University in Rabat and the University of Pennsylvania in Philadelphia over nine years, from 1981 to 1990. Daniel Wagner begins his book based on this study (Wagner 1993) with a vignette of Oum Fatima, an older woman living in one of their research sites:

On some days, the mailman would arrive with letters: Oum Fatima would deliver each to the addressee, knowing simply by the type of handwriting or script used – Arabic or French – who should receive which letter … At the *souk* (market), Oum Fatima’s skill in mental arithmetic and bargaining was legendary. Not only could she switch effortlessly between the several parallel currency units in use … but her ability to negotiate the lowest possible price made her a well-known figure in the *derb* (quarter). To those of her social class, as well as to those “higher up”, Oum Fatima was a woman of great respect (Wagner 1993, p. 1).

The account gives a rich insight into Oum Fatima’s abilities, including how she could make calculations easily and spontaneously between different currencies and numeracies – “dirhams, francs and riyals (a base-five system)” (ibid.). Wagner goes on to say that “[w]hether Oum Fatima is illiterate or literate or somewhere in between depends on one’s frame of reference” (ibid., p. 3), commenting that although local educators and policymakers would consider her “illiterate”, people in her neighbourhood “consider her … among the most competent people they know” (ibid., p. 2). What is interesting here – and of particular relevance to discussions on literacy and citizenship – is Oum Fatima’s confidence in engaging with written texts and numeracies, without any formal schooling. This not only demonstrates her knowledge and confidence, built up through her role as an active citizen in this community, but also that she has learned informally how to conduct complex numerical transactions and even how to recognise different written scripts.

Through the lens of an ideological model of literacy – also discussed as a “social practice” or “situated” approach to literacy (and numeracy) – this example challenges the idea of a divide between literate and illiterate, literacy and orality. Instead, Street (1993) proposed a continuum, and introduced the concept of multiple literacies and multiple numeracies. Applying this understanding of literacy, rather than a notion of literacy as “schooled” and consisting of the kind of reading and writing practised in formal educational institutions (Street and Street 1991), helps shift attention on to what women like Oum Fatima *can* rather than cannot do – and to recognise the competencies that her neighbours respect. Adopting this perspective on literacy in relation to citizenship education challenges educators to move away from a deficit perspective on learners, to consider instead how and when they engage with literacy practices in their everyday lives. What is of particular relevance to literacy planners is exploring how informal learning connects with formal literacy instruction.

I will turn now to two recent ethnographic studies to investigate the connection between literacy and citizenship through a social practice lens – though, significantly, neither of them looks at literacy *for* citizenship. Ahmmardouh Mjaya (2022) explores the experiences of women participating in a government-run literacy programme in a Malawian rural community and analyses the meanings of literacy to them and others. Ms Awala, one of the adult literacy participants, explained to him that she was not interested in acquiring a certificate from her literacy class:

“I just go there to make sure that I master my name so that when we are called for some other activities, I should be able to sign using a pen. I have already started doing this; even when we … went to receive money to buy fertilizer, I got hold of the pen, and they said ‘Grandma, are you going to sign?’ I said ‘yes’. They said ‘we respect you!’” (ibid., p. 68).

In this place, the alternative to signing one’s name on receipt of various development inputs was to give a thumb print. Ms Suya, another participant, described how officials seeing an older woman automatically got out the inkpad – “they grab our hands and make us print using our thumbs” (ibid., p. 69). In this example, the hierarchies of meaning-making are clear and related to identity and status, as Mjaya observes:

While the pen symbolized literacy and somehow raised the confidence and status of those who could get hold of it, the inkpad symbolised “illiteracy”, thereby making those who pressed their thumbs on it as a way of signing feel public shame and humiliation (ibid., p. 68).

For Ms Awala, learning how to write her name in the literacy class was not just about gaining a functional skill but also around gaining respect from others in the community, particularly from formally educated people. The symbolic meanings associated with literacy here were valued by Ms Awala and her classmates and perhaps drew them to enrol in a literacy class. Though Mjaya’s research revealed that non-literate women also found ways to vote in committees and work as traditional leaders with help from younger educated women who acted as literacy mediators, some older women were keen to be able to perform such tasks involving reading and writing independently

In the Philippines, Christopher Millora (2020) conducted ethnographic research with an informal settlers’ association fighting for land tenure, led by landless volunteers. He investigated many examples of “bureaucratic literacies” which the volunteers had to engage with through their activism – complex processes and texts linked with land registration, accounts and running the organisation. For instance, the treasurer of the association, Mila, explained how she did the financial statement:

“I just thought of it. I asked myself how I can liquidate the money, one by one, so it would also be easy for people to understand. Susan taught me at the start what to do … but I wasn’t comfortable, it was difficult to do … so I just did it like this so that if the auditor reads, it’s easy” (Millora 2020, p. 147).

Mila’s account gives an insight into how she learned to do the accounts “on the job”, through peer instruction and initial support from her colleague Susan, then working out a system that seemed simpler and more straightforward. In contrast to the Malawi extract, this is an example of functional literacy and numeracy skills linked to citizenship and learned in everyday life, “on the job”, rather than in a classroom. Mila has the confidence to “take hold” (Kulick and Stroud 1993) of this literacy practice through her desire to make the financial statement more accessible to her peers.

These examples from Malawi and the Philippines show the importance of conceptualising literacy in relation to social practice, as shaped by social relationships and power. Rather than regarding literacy as only the technical skills of decoding letters and symbols, we need to look at what it means in a specific social context – embedded or situated literacy. In both these situations, the women were challenging power relations through literacy – Ms Awala through the symbolic act of signing her name and Mila through performing and adapting literacy/numeracy practices associated with high-level bureaucratic processes. Both examples illustrate that literacies, numeracies and multimodal communicative practices (such as the thumb print) are not only diverse and multiple, but also hierarchical – and such research has implications for literacy and citizenship education. Mjaya’s study reveals how everyday life shapes what people take from the classroom – and why they enrol in literacy classes in the first place. In this case, it was the symbolic act of signing with a pen rather than a thumb print, rather than any specific functional literacy skills, which they valued. Through focusing on how people engage with, mediate and learn literacy and numeracy practices in running an organisation, Millora gives insights into informal learning of bureaucratic literacies.

Relating these ideas to my earlier review of citizenship education, learning literacy here is very much around social change, empowerment and engaging with new identities. This connects with the concept of global citizenship education as “transformative” (Hanemann 2019; UIL 2022a), and contrasts with the more instrumental notion of literacy within earlier citizenship discourses, as preparation for learning and practising civic duties. I suggest that education providers and policymakers could build on the informal and spontaneous literacy learning that people engage in through everyday citizenship activities, and actively explore meanings of literacy in specific communities – if such a process is to be empowering.

## Exploring the implications for literacy programmes

I will conclude this article by looking at the pedagogical implications of applying the theoretical lenses introduced above to literacy and citizenship. Since it is beyond the scope of this article to review case studies of best practice in literacy programmes, my aim here is to identify some key features and principles that could enable educational providers to strengthen the connections between literacy and citizenship.

First, ethnographic research on everyday literacies has revealed ways in which “citizenship is caught not taught” (Davies 2012, p. 37). Active citizenship has often been talked about as a useful way of introducing participatory methods into the classroom. But the above examples suggest that it is also about how people learn informally as active citizens in real life – whether Oum Fatima bargaining in the *souk* or Mila maintaining her association’s financial records. As educators, we need to find ways of strengthening the connections between classroom and everyday situations where literacy for citizenship plays a role. All too often, programmes have taken a “literacy first” approach (Rogers 2000) – where the assumption is that adults will become more active citizens if they are first encouraged to read about their rights in a textbook in a literacy classroom. By contrast, a “literacy second” approach focuses on ways of facilitating literacy learning through a real-life activity – in this case, an activity related to citizenship such as keeping accounts or filling in land registration forms. Functional literacy skills for citizenship – as Mila’s example shows – can be taught and learned effectively through actually practising citizenship. The “real literacies” approach (Rogers et al. 1999) has been promoted as a way of supporting the literacy and numeracy activities that participants engage with in their lives outside the classroom. Rather than teaching from examples in a textbook, this involves the facilitator and students bringing “real” texts into the literacy classroom, such as, for example, voting slips, election posters or online registration forms. Structured lessons can then be constructed around these texts and practices – or alternatively, some educational providers have set up “drop-in” centres where people can come for support with documents that they need to fill in.

Second, digital literacy is now central to our lives, and Freire’s critical pedagogical approach has even more relevance to our engagement as active citizens with digital communication – as Devina Sarwatay et al. suggest, “[a]s more of our existence is being digitally datafied, citizenship itself is being digitised” (Sarwatay et al. 2021, p. 6). Research on citizenship education in schools has revealed that information and communications technology (ICT) is more often seen by policymakers and politicians, as John Naughton (1998) pointed out, “as a kind of a pipe for pumping information into schools and schoolchildren” (cited in Haydn 2012, p. 172). In adult literacy classrooms and agricultural or health extension programmes, similar limitations can be seen where, for instance, mobile phones have been used to convey useful information through text messages – rather than educators and adult literacy participants engaging with ICT as a medium of active communication and creative way of constructing knowledge. Within this approach, adult participants have tended to be situated as consumers rather than co-creators of knowledge and literacies – which could be seen as the opposite of “active citizenship”.

Abeer AlNajjar (2019) observes that much policy discourse focuses only on the risks of digital media, rather than their potentially positive role in enhancing “learning, self-expression and the social good” (ibid., p. 77). Writing on critical media literacy in the Middle East, she argues the urgency to adopt a “pro-active approach to prepare youth for critical and informed engagement with the ‘network society’” (ibid., p. 74, referring to Castells 1997). She points out that young people are constantly navigating online spaces in their everyday lives “that are over-saturated with content, highly politicized, volatile and polarized” (ibid.). Therefore, there is a need to focus on young people’s voices, agencies and identities in relation to digital engagement as citizens. In the context of the Arab Spring, for instance, young activists who spoke English were better able to advance their digital literacy skills through informal learning in online spaces. In this case, the issues of inequality in seizing opportunities for active citizenship were not only around access to ICT, but also access to English language – and have important implications for educational providers. AlNajjar explains how learning to understand “the sender’s political and social agenda” (ibid., p. 75), for instance, can help young people to engage more critically with social media. With the proliferation of (mis)information about cures for and causes of COVID-19 during the pandemic, this kind of approach to critical digital literacy seems even more essential to our roles as active citizens.

Finally, global citizenship education goes beyond teaching functional literacy for civic education within adult literacy and citizenship programmes. A commitment to transformative global citizenship and recognition of citizenship as based on multiple identities points to the importance of valuing Indigenous languages, knowledges, learning and literacies. The challenge is how to create spaces for sharing Indigenous approaches to learning and literacies within more formalised programmes of instruction – without undermining “alternative” pedagogies and knowledges. This goes back to Nussbaum’s concept of the “narrative imagination” (Nussbaum 2002, p. 299) within world citizenship education, where the focus is on empathy and understanding other people’s values from outside one’s own group/society – “prepar[ing] students to understand the situation of people different from themselves” (ibid., p. 300). The best fictional writing allows the reader to do just this, through entering someone else’s world, emotions and thinking – a very different experience from reading the didactic texts that often form the backbone of adult literacy and citizenship programmes. This is where learner-generated materials and language experience approaches to adult literacy programmes – based on sharing life stories orally through written text in local languages – might help provide an important first step into creative writing and reading. The starting point for developing empowering and transformative literacy and citizenship programmes needs to become a recognition of people’s experiences, aspirations and identities – rather than aiming only to introduce and induct them into dominant literacy and citizenship practices.

## Conclusion

Although citizenship education has emerged as a much broader endeavour than teaching skills for civic engagement, literacy has tended still to be seen in the narrow terms of the earlier paradigm. This has led either to teaching people how they should be living their lives through reading stories about “good citizenship”, or teaching functional literacy skills for citizenship divorced from real-life situations. In programmes where citizenship is seen in terms of Freire’s “conscientisation” (Freire 1996 [1970]), literacy is often used synonymously with “knowledge” and there has been little attention to exploring the ways in which people already use and want to engage with literacy in their everyday lives. Reflecting on the feminist critique of citizenship education as being focused on the male domain (Arnot and Dillabough 2000), the dominant “literacy first” approach (Rogers 2000) appears to have centred on introducing literacy skills for public forms of governance and representation. Mjaya’s recent study in Malawi (Mjaya 2022) points to the importance of investigating gendered identities in relation to literacy and citizenship in everyday spaces – rather than assuming that literacy practices carry the same meaning for women and men in a certain community.

Current approaches to literacy instruction suggest a need to engage more creatively with the concepts underlying global citizenship education – particularly the understanding of our “shared fate” (Williams 2003, p. 209), development of “narrative imagination” (Nussbaum 2002, p. 299) and valuing multiple identities. In its *GRALE 5 Executive Summary,* UIL emphasises the importance of nurturing empathy and connections through citizenship education:

Target 4.7^2^ suggests that the way to balance the needs of planet, people and prosperity is by fostering, through education, the kind of citizenship that will allow our most truly human and humane values – of love, care and responsibility – to emerge (UIL 2022b, p. 11).

The role of literacy within this kind of citizenship education is not only to provide skills for representation, documentation and accountability, but also to bring people closer together by sharing experiences, values, voices and aspirations and facilitating deeper interactions through written, oral and digital texts.

The relationship between literacy and citizenship is often presented unproblematically as being literacy *for* citizenship, underpinned by an assumption of a causal relationship between enhanced literacy skills and active citizenship. By developing an exploration of literacy and citizenship based on a theoretical model of literacy as a social practice, I have focused on literacy practices in everyday life rather than only in the classroom. This is not just about considering how literacy for citizenship can be learned (whether formally or informally) through engaging with “real literacies” – such as learning to read voting slips – but also about using this theoretical lens to investigate the ways in which literacy is shaped by social relationships in everyday life. Thus this article moves beyond literacy *for* citizenship to considering the ways in which literacy learning can emerge *through* active citizenship. Introducing the notion of multiple and multimodal literacies can help to broaden and deepen understanding of hierarchies of literacies and languages in relation to people’s multiple and changing identities. Rather than starting from an assumption that people have one dominant identity which shapes their aspirations and rights as citizens, this stance focuses our attention instead on intersectionality and diversity. Significantly, this alternative theoretical perspective on literacy and citizenship raises different questions – including asking whether literacy is and should be a prerequisite for active citizenship.

## Footnotes

1 The historical context is important here too – in the 1970s, only literate adults in Brazil had a vote. So being literate carried very concrete advantages in relation to citizenship. See Freire and Macedo (1998, p 20).

2 Within the fourth Sustainable Development Goal (SDG 4) of the United Nations 2030 Agenda, Target 4.7 aims to “ensure that all learners acquire the knowledge and skills needed to promote sustainable development, including, among others, through education for sustainable development and sustainable lifestyles, human rights, gender equality, promotion of a culture of peace and non-violence, global citizenship and appreciation of cultural diversity and of culture’s contribution to sustainable development” (UN n.d.).
